# Target Molecular Simulations of RecA Family Protein Filaments

**DOI:** 10.3390/ijms13067138

**Published:** 2012-06-11

**Authors:** Zhi-Yuan Su, Wen-Jay Lee, Wan-Sheng Su, Yeng-Tseng Wang

**Affiliations:** 1 Department of Information Management, Chia Nan University of Pharmacy & Science, No. 60, Sec. 1, Erren Rd., Rende Dist., Tainan City 71710, Taiwan; E-Mail: zysu@mail.chna.edu.tw; 2 National Center for High-Performance Computing, Hsin-Shi, No. 28, Nan-Ke 3rd Rd., Hsin-Shi Dist., Tainan City 74147, Taiwan; E-Mails: wjlee@nchc.narl.org.tw (W.-J.L.); c00ccu00@nchc.narl.org.tw (W.-S.S.); 3 Department of Physics, National Chung Hsing University, No. 250, Kuo Kuang Rd., Taichung 402, Taiwan

**Keywords:** molecular dynamics, homology modeling, archaeal rada, DNA SOS repair process, ATP binding proteins

## Abstract

Modeling of the RadA family mechanism is crucial to understanding the DNA SOS repair process. In a 2007 report, the archaeal RadA proteins function as rotary motors (linker region: I71-K88) such as shown in Figure 1. Molecular simulations approaches help to shed further light onto this phenomenon. We find 11 rotary residues (R72, T75-K81, M84, V86 and K87) and five zero rotary residues (I71, K74, E82, R83 and K88) in the simulations. Inclusion of our simulations may help to understand the RadA family mechanism.

## 1. Introduction

**Figure 1:**
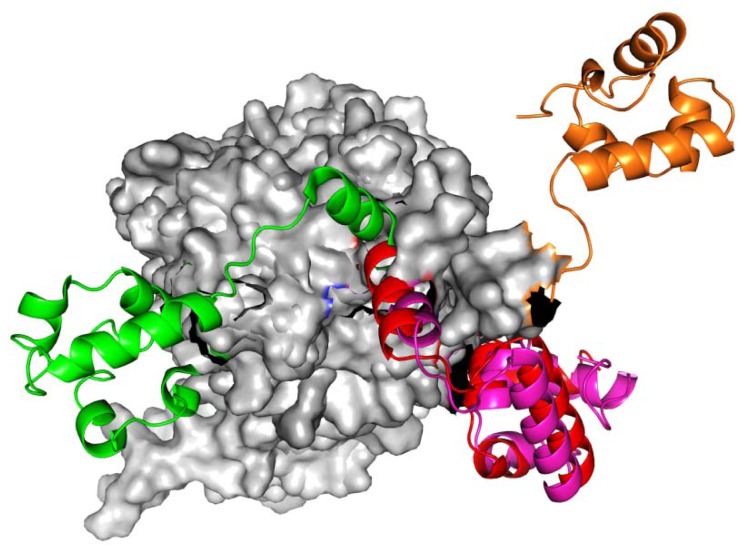
The RadA rotary motor model is from left-hand RadA filament (green) to closed-ring RadA filament (red), then to MvRadA filament (pink), then to right-hand RadA filament (orange) and finally to left-hand RadA filament (green). The *N*-terminal domain (NTD) is indicated in gray.

Homologous recombination is a process whereby two DNA duplexes interact to transfer genetic information, then create new genetic linkages and rearrange DNA segments. Genes involved in homologous recombination are important for regulation of gene expression and DNA repair [1,2]. In the recombination process, RecA-like proteins can bring two homologous DNA molecules together and exchange the DNA strands [2–4]. Examples of RecA-like proteins include archaeal RadA, bacterial RecA, Rad51, meiosis-specific Dmc1 and eukaryotic Rad51. At DNA double strand (dsDNA) break sites, these proteins interact with single-stranded DNA (ssDNA) and form a right-handed helical nucleoprotein filament (presynaptic complex) [5]. The presynaptic complex has both ATPase and DNA strand exchange activities. In the presence of ATP molecules, the DNA strand exchange activity ensures the formation of heteroduplex DNA (hDNA) between ssDNA and its complementary strand in the double-stranded DNA (dsDNA). The molecular mechanism has been proposed such that ATP hydrolysis promotes product release and a rotary mechanism that can solve the DNA topological problem in the strand exchange reaction [6–8]. In terms of structural functionalism, RecA proteins have similar *N*-terminal domains (NTD) that may interact with dsDNA, and the *C*-terminal domain that may hydrolyze ATP molecules and form the new conformation [9–11]. RecA proteins may form closed rings, as well as left-handed protein filaments and right-handed helical filaments [12–17]. The molecular mechanism underlying the conformational flexibility of RecA proteins is still unclear. In a 2007 report [17], Wang *et al*. proposed that: (1) right-handed RadA proteins will interact with ssDNA and form presynaptic complexes; (2) ATPase of presynaptic complexes decomposes ATP molecules (ATP→ADP). Then the hydrolysis energy can force the DNA strand exchange; (3) the complexes might release the ADP molecules and prepare to dissociate the DNA strands; and (4) after dissociating the DNA strands, left-handed RadA proteins might be formed. The RadA proteins might function as rotary motors (linker region: I71-K88) such as the mechanism shown in Figure 1.

MD techniques can offer a convenient alternative to experimental approaches because they can treat a single macromolecule at an atomic level. TMD (target molecular dynamics) [18] methods can provide calculated reaction paths for most proteins by continuously decreasing the target values. This method can predict reaction paths of *ras* p21 proteins [19] and chymotrypsin inhibitor 2 proteins [20]. A TIP3P solvent model allows all of the archaeal RadA protein’s rotation trajectories to be sampled.

The cumulative changes in the backbone dihedral angles (CCDA) method can predict important residues in biomolecular dissociation systems [21–26]. The backbone dihedral angles of proteins are called phi (ϕ, involving the backbone atoms C′-N-Cα-C′) and psi (ψ, involving the backbone atoms N-Cα-C′-N). Thus, phi controls the C′-C′ distance and psi controls the N-N distance. CCDA is defined as:

(1) $${CCDA}_{\left(\alpha \right)}=\Sigma {\left(\alpha \right)}_{j}$$

Here, α is the phi or psi angle, and j is residue number. In biomolecular systems, backbone rotations are provided with higher energy barriers, and backbone dihedral angles are representative of backbone rotations [27,28]. Thus, counting the cumulative changes in backbone dihedral angles can predict important residues of the Abs-Ag complex.

In the present study, we used the Sulfolobus solfataricus (Sso) protein sequence and related 3D structures (closed-ring RadA, MvRadA, right-hand RadA and left-hand RadA) [12,17,29,30] to normalize the 3D archaeal RadA structures. Then the TMD method was used to investigate the conformational mechanism of the archaeal RadA proteins. We also reveal the conformational mechanism, the CCDAs (cumulate changed dihedral angles) of rotational residues (I71-K88), the system’s potential energy, and RMSD of archaeal RadA proteins in the simulation process.

## 2. Results and Discussion

### 2.1. Homology Models Construction and Evaluation

**Figure 2:**
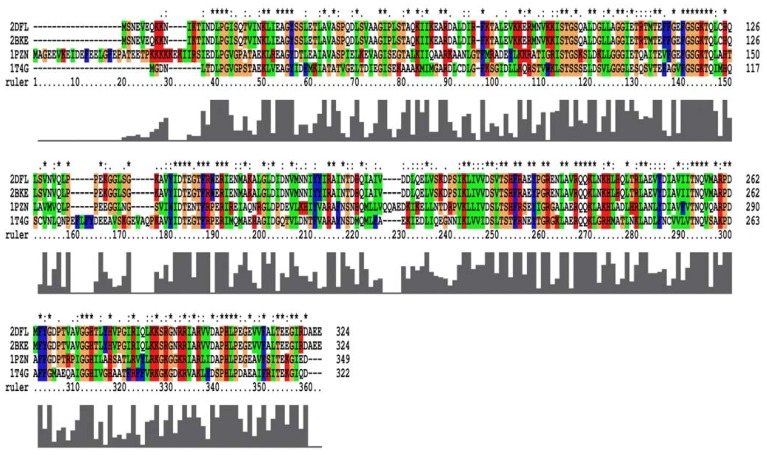
The multiple sequence alignment for the four RadA protein filaments. 2DFL is the left-hand RadA filament, 2BKE is the right-hand RadA filament. 1PZN is the closed-ring RadA filament, and 1T4G is the MvRadA filament.

From the 2007 report [17], the closed-ring, MvRadA and right-hand RadA proteins can be used as potential templates for homology models. For closed-ring, MvRadA and right-hand RadA proteins, the percentage of sequences identifying with the left-hand RadA protein sequence are 51.6%, 44.0% and 100% (Figure 2). From Prosa method validations, the *z*-score of the four proteins are −8.91, 0.09, −2.47 and −7.86 for closed-ring, MyRadA, right-hand RadA and left-hand RadA, respectively. These initial structures are a good starting point for building reliable models in the next step.

**Figure 3:**
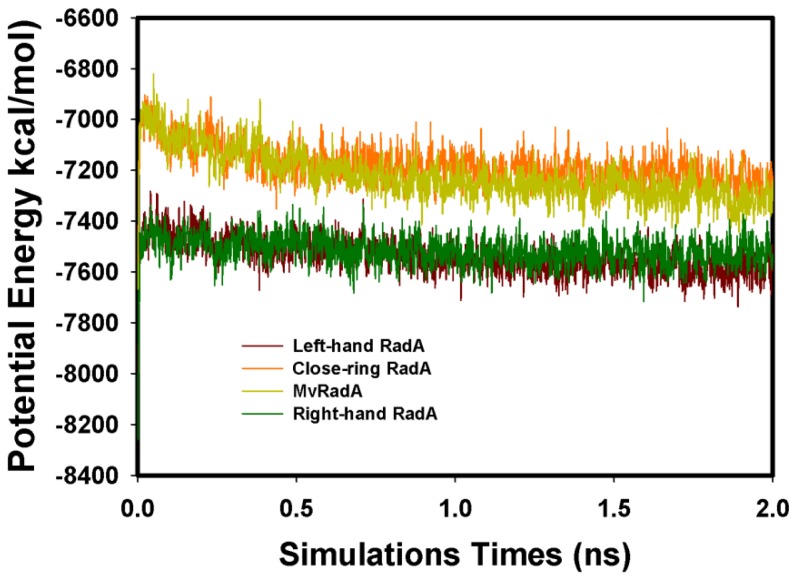
The total MD simulation energy profiles of the four RadA filaments.

The four initial proteins were refined by energy minimization and MD simulations. Figure 3 displays the total energy *versus* frames collected per 1ps during the entire 2 ns MD simulation. Clearly, the total energies of four systems were equilibrated after 0.3 ns. The structures were set as the final models by 2 ns simulations. Final models of the four proteins were assessed by PROSA and PROCHECK. By Prosa method validations, the *z*-scores of the four proteins are −8.95, 0.01, −2.53 and −7.97, respectively. As to the assessment by PROCHECK, the reliability backbone torsion angles of the four RadA filaments were examined. In the core Ramachandran region, the percentage of dihedral angles is 100.0%, 98.8%, 98.6% and 100.0% for left-hand, closed-ring, MvRadA and right-hand RadA proteins, respectively. The data indicate that these 3D models are reliable for further TMD simulations.

### 2.2. TMD Conformational Calculations

**Figure 4:**
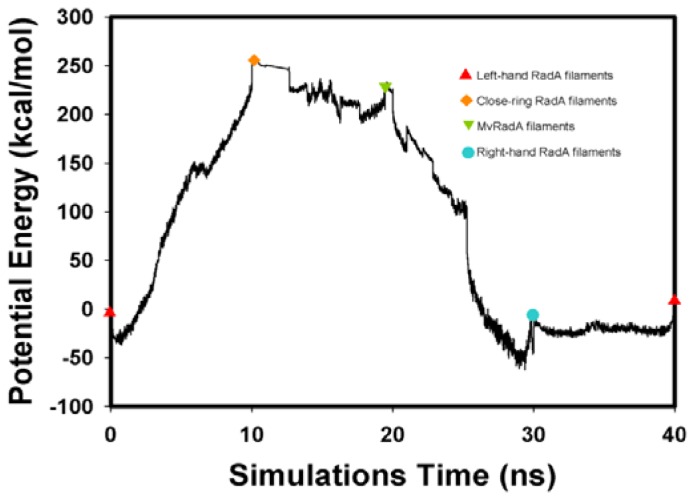
The energy profiles of the RadA filament simulations process from TMD calculations. 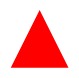, Left-hand RadA filaments; 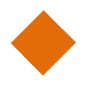, Close-ring RadA filaments; 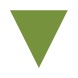, MvRadA filaments; 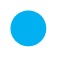, Right-hand RadA filaments.

**Figure 5:**
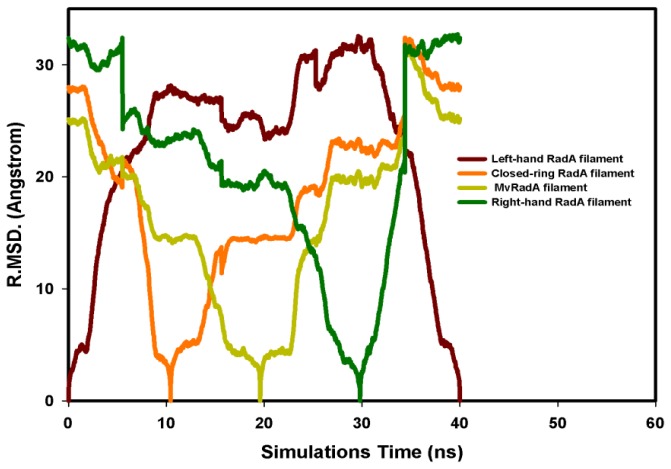
The RMSD profiles of the 40 ns TMD simulations. (**A**) Left-hand RadA is indicated in brown; (**B**) Closed-ring RadA is indicated in orange; (**C**) MvRadA is indicated in light green; (**D**) Right-hand RadA is indicated in dark green.

The potential energy of our left-hand filament is reset to zero, with the energy profiles of the TMD calculations shown in Figure 4. The four potential energy states are 0.00, 254.14, 223.54 and −10.16 kcal/mol. The results in Figure 4 indicate that two energy barriers (0–28 ns and 28–33 ns) occur in the 40 ns simulations processes. Analyzing the energy profile, we find that the major energy barrier occurs during the conformational change between left-hand and pre-right-hand proteins. The major energy barrier height is approximately 280.00 kcal/mol. The minor energy barrier exists as the conformation turns into the right-hand protein. The energy barrier height is approximately 60.00 kcal/mol. The TMD simulation trajectories of the calculations were traced by RMSD and CCDAs methods. Figure 5 shows the 40 ns TMD simulation profiles of RMSD. Due to the simulation’s initial structure of RadA filament (left-hand), the profile of the left-hand RadA filament indicates that the RMSD will increase 27 Å within 10 n, and the value will fluctuate between 25 and 35 Å within 10–30 ns, with the value decreasing to 0 Å at 30–40 ns. The other RMSD profile shows the RMSD values for closed-ring, MvRadA and right-hand RadA filaments.

**Figure 6:**
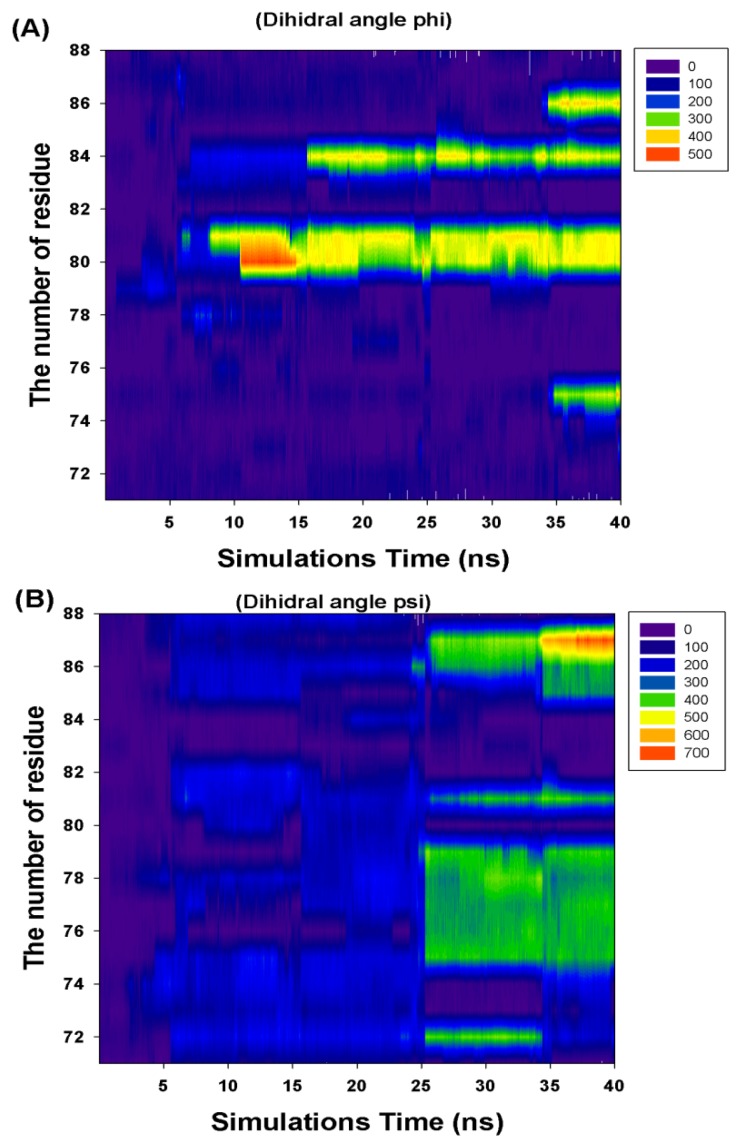
The profile of the rotational residues’ (I71-K88) cumulate changed dihedral angles (phi and psi).

Figure 6 and Table 1 show the CCDAs of the rotational residues (I71-K88). The results indicate that the CCDAs of the five residues (I71, K74, E82, R83 and K88) are zero in the simulations.

Analyzing the major barrier (0–28 ns), we find that the 11 variations (R72, T75-K81, M84, V86 and K87) are obvious in the CCDAs. At 0–10 ns, the system tends to fold the closed-ring RadA (Figure 5) and the system energy rises to 254.14 kcal/mol. The K81 is the obvious CCDA variation and the event occurs at 6 ns. At 10–20 ns, the system conformation exists between closed-ring and MvRadA proteins. The system energy decreases to 223.54 kcal/mol and three variations (K80, K81 and M84) are obvious in the CCDAs. The K80 variation occurs at 12 ns and the other variations occur at 17 ns. At 20–28 ns, the system energy decreases to −70 kcal/mol and four variations (R72, T75, A76 and E78) emerge at 25 ns in the CCDAs. Analyzing the minor barrier (28–33 ns), we find that the obvious variations of CCDAs are the same as for the major energy barrier analysis.

## 3. Experimental Section

### 3.1. Homology Modeling and Refinement of the Models

Homology modeling is a computational approach for three-dimensional protein structure modeling and prediction. Proteins whose structures are still uncharacterized can be built using homology modeling. This method builds an atomic model based on experimentally determined known structures that have sequence homology of more than 40% with the target. Modeling structures with less than 40% template similarity would result in a less reliable model. Homology modeling is also known as comparative modeling. To normalize the 3D archaeal RadA structures, the homology modeling method was applied in the study. All the primary protein sequences and structures can be obtained from the protein data bank [12,17,29,30]. For the four target sequences (closed-ring RadA, MvRadA, right-hand RadA and left-hand RadA), the multiple sequence alignment was calculated by ClustalX2 program [31]. According to the left-hand RadA protein sequence and the template structures (PDB ID: 1PZN and 1T4G), the closed-ring RadA and MvRadA structures were constructed by the Modeller program [32]. The disappearing L2 region [33] of the right-hand RadA (M258-H275) was constructed by the loop optimization method and very slow loop refinements method found in the Modeller program.

The structural refinement process was accomplished in several stages. First, energy minimization of 10,000 steps steepest decent (SD) followed by 30,000 steps conjugated gradient (CG) was carried out while fixing the backbone Cα. Then, we generated the solvent box (TIP3 water molecules and RadA protein) of volume 89.27 × 81.69 × 88.17 Å^3^. The initial simulation system combined the RadA protein and the solvent box. The water molecules around the RadA protein with 2 Å were deleted. The total number of water molecules was 6104 and the total number of atoms in the system was 53,081. After performing 20,000 step CG energy minimization, MD simulations were performed with 2 ns using an NVT ensemble, periodic boundary conditions box (89.27 × 81.69 × 88.17 Å^3^), and particle mesh Ewald (PME) method at a temperature of 310 K. The energy minimization and MD simulations mentioned above were accomplished by the Amber program and force fields [34]. The final structures were checked by PROCHECK [35] and PROSA [36].

### 3.2. TMD Simulations

An additional energy term based on the RMSD of the RadA proteins is relative to a prescribed target structure. The energy term (*U_TMD_*) is defined as the formula:

(2) $$U_{TMD}=\frac{1}{2}\frac{K}{N}{\left(RMSD(t)-{RMSD}_{0}(t)\right)}^{2}$$

where the force constant, *K*, is 200 kcal·mol^−1^·Å^−2^. *N* is the number of target atoms. *RMSD(t)* is the RMSD of the simulation structure at time *t* relative to the prescribed target structure, and *RMSD_0_(t)* is the prescribed target RMSD value at time *t*. As described above, four TMD simulation cases were simulated in the study. The four cases were left-hand/close-ring, close-ring/MvRadA, MvRadA/right-hand and right-hand/left-hand RadA proteins. The target proteins were close-ring, MvRadA, right-hand and left-hand RadA protein, respectively. The TMD simulations were calculated with the NAMD [37] software and 10 ns simulation time.

## 4. Conclusions

In this article, we propose using molecular simulations techniques to analyze the conformation mechanism of RadA proteins. We report the two energy barriers, 11 high rotational residues and 5 zero rotational residues (I71, K74, E82, R83 and K88) in the TMD simulations. If the initial conformation starts as the left-hand RadA protein, the system should overcome the two energy barriers and the 11 residues (R72, T75-K81, M84, V86 and K87) to provide high conformation degree of freedom. The high rotational residues might increase the flexibility of RadA and make the conformation change easy. The zero rotational residues are the axis of the conformation change mechanism. In the next phase of the project, we will use molecular biology methods to mutate the residues included 11 high rotational residues and 5 zero rotational residues. We hope this finding might help to understand the RadA family mechanism.

## Figures and Tables

**Table 1:** The cumulate changed dihedral angles (I71-K88) of the rotational residues.

Residue Number	Cumulate Changed Dihedral Angles	

	Phi (Φ)	Psi (Ψ)
I71	0	0
R72	0	360
F73	360	0
K74	0	0
T75	360	360
A76	0	360
L77	0	360
E78	0	360
V79	0	360
K80	360	0
K81	360	360
E82	0	0
R83	0	0
M84	360	0
N85	0	360
V86	360	360
K87	0	720
K88	0	0
